# Synergizing radiotherapy and immunotherapy for locally advanced gastric cancer: evolving paradigms and future directions

**DOI:** 10.3389/fimmu.2026.1875451

**Published:** 2026-06-26

**Authors:** Shuhui Lin, Wenji Pu, Xiaoye Su, Junqin Lei, Juan Li, Chen Chen, Jin Xu, Qin Xiao, Zicheng Zhang, Jing Jin

**Affiliations:** 1 Department of Radiation Oncology, Shenzhen Nanshan People’s Hospital, Shenzhen, China; 2 Department of Radiation Oncology and Shenzhen Proton Therapy Center, National Cancer Center/National Clinical Research Center for Cancer/Cancer Hospital & Shenzhen Hospital, Chinese Academy of Medical Sciences and Peking Union Medical College, Shenzhen, China; 3 Medical Department of Shenzhen University/General Hospital of Shenzhen University/Academy of Clinical Medicine of Shenzhen University, Shenzhen, China; 4 Department of Radiation Oncology, South China Hospital, Medical School, Shenzhen University, Shenzhen, China; 5 State Key Laboratory of Molecular Oncology and Department of Radiation Oncology, National Cancer Center/National Clinical Research Center for Cancer/Cancer Hospital, Chinese Academy of Medical Sciences and Peking Union Medical College, Beijing, China

**Keywords:** gastric cancer, gastroesophageal junction adenocarcinoma, immune checkpoint inhibitors, neoadjuvant chemoradiotherapy (NCRT), precision oncology, radiotherapy

## Abstract

This article innovatively reviews and unveils the synergistic mechanisms, clinical research directions, and future challenges of the combination of preoperative radiotherapy (RT) and immunotherapy (especially the most popular belonging to immune checkpoint inhibitors, ICIs) in the treatment of locally advanced gastric cancer and gastroesophageal junction adenocarcinoma (GC/GEA). The integration of RT and ICIs represents a promising therapeutic strategy for locally advanced, even unresectable, GC/GEA. RT potentiates antitumor immunity by inducing immunogenic cell death (ICD) and targeting iron death, activating the cyclic Guanosine Monophosphate (GMP) and Adenosine Monophosphate (AMP) synthase-stimulator of interferon genes (STING protein) (cGAS-STING) signaling pathway, enhancing the expression level of the major histocompatibility complex (MHC) molecule and immune checkpoint proteins on tumor cells, and promoting immune cell infiltration into the tumor micro-environment. Trials with small sample sizes, such as Neo-PLANET and SHARED, have demonstrated that neoadjuvant chemoradiotherapy (NCRT) combined with ICIs yields encouraging pathological complete response (pCR, ranging from 22.6% to 38.2%) and high R0 resection rates with manageable toxicity profiles. Nevertheless, conflicting results from phase I-II trials like ECOG-ACRIN EA2174 underscore the necessity for patient stratification based on robust biomarkers. Current evidence regarding tumor cell programmed cell death protein ligand 1 (PD-L1) expression (namely, PD-L1 combined positive score or tumor proportion score), tumor mutational burden (TMB), and intratumoral immune micro-environment features for identifying responders still remains inconclusive. Future efforts should prioritize the validation of predictive biomarkers (containing the cutting-edge ctDNA), RT dose, and target area definition (especially for primary positive tumors and high-risk lymphatic drainage); optimization of RT-ICIs sequencing; and the conduct of large-scale randomized controlled trials to establish survival benefits and standardize combination protocols according to the stratified population.

## Highlights

- Radiotherapy (RT) synergizes with immune checkpoint inhibitors (ICIs) to enhance anti-tumor immunity in locally advanced gastric/gastroesophageal junction adenocarcinoma (GC/GEA) through inducing immunogenic cell death (ICD) and ferroptosis, cGAS–STING pathway activation, and increased immune cell infiltration.
- The application of RT in the perioperative period is undergoing reevaluation: from adjuvant to neoadjuvant efficacy. Recent phase I-II trials (Neo-PLANET, SHARED, et al.) demonstrate promising pathological complete response (pCR, an increase in pCR rate from 22.6% to 38.2%) and R0 resection rates with acceptable toxicity for neoadjuvant chemoradiotherapy (NCRT) combined with ICIs but consistently lack long-term survival outcomes.
- Predictive biomarkers such as tumor cell programmed cell death protein ligand 1 (PD-L1) expression, baseline tumor mutational burden (TMB), and immune micro-environment features show potential but require further validation for reliable patient stratification. Perioperative decision-making guided by biomarkers including circulating tumor DNA (ctDNA) may further advance the development of GC treatment strategies.
- Optimal integration of preoperative RT and immunotherapy-containing therapies (namely emerging CTLA-4/PD-(L)1 blockade, lacking convincing phase II/III data in the neoadjuvant RT setting)—including RT technique selections (IMRT, VMAT, or proton owing to the “Bragg Peak”), segmentation dosing and modalities (like combining low-dose radiotherapy and stereotactic body radiotherapy), target volume definition (regional lymphatic sparing strategy), RT intervention timing, and delivery sequencing (e.g., anti-PD-1/PD-L1 treatment scheme during the RT)—remains controversial for balancing maximizing efficacy and minimal adverse events (AEs) in the field of GC/GEA.
- Large-scale randomized controlled trials (RCTs) are urgently needed to confirm survival benefits and establish standardized combination protocols stratified according to tumor characteristics (such as the tumor’s primary location and subtypes, genetic typing, and spatial immune infiltration map) and biomarker-enriched subgroups for locally advanced or even unresectable (aiming to transform) GC/GEA.

## 1 Introduction

Gastric cancer and gastroesophageal junction adenocarcinoma (GC/GEA) rank as the 5th most common malignant tumor globally, with approximately 968, 000 new cases reported in 2022, and are the 5th leading cause of cancer-related deaths (1). Early-stage GC has a relatively favorable prognosis, with a 5-year overall survival (OS) rate of up to 95% through multidisciplinary or multimodal treatments, including surgery, adjuvant chemotherapy (chemo), and complementary radiotherapy (RT) for high-risk groups (2, 3). However, due to the nonspecific nature of early symptoms, approximately two-thirds of patients are diagnosed at a locally advanced or even metastatic stage when symptoms become evident, especially in China and the Orient (representative of Japan and South Korea). On the one hand, the aforesaid perioperatively conventional therapies offer poor prognostic improvement for resectable GC/GEA. The reason is that RT can effectively “clear the local battlefield” (improving pCR), but it is powerless against tumor cells or circulating tumor DNA (ctDNA) hidden in the blood or peritoneum (early tendency for hematogenous/peritoneal metastasis), which is the root cause of short-term recurrence (4). On the other hand, GC is highly heterogeneous and lacks clear targets for RT and chemo. Among them, diffuse type and genomically stable type are naturally insensitive to traditional therapy. Excitingly, immunotherapy functions by activating the host’s immune cells to recognize, target, and eliminate tumors, thereby reducing the residual tumor burden in the body. Hence, the outstanding effectiveness of immune checkpoint inhibitors (ICIs) is known as the “third revolution” in anti-tumor treatment and has gained much attention from the investigators at home and abroad, especially its synergism with RT.

## 2 Historical evolution of RT for GC/GEA

### 2.1 The irreplaceable role of radiation

Prior to the advent of immunotherapy, RT firmly played a key role as a primary treatment modality for GC/GEA, contributing to not only increased pCR rate (about 15.6%-23%) and R0 resection rate but also reduced local recurrence risk, as evidenced by internationally recognized studies such as CROSS and POET (5, 6). According to long-term follow-up results, these advantages ultimately translate into survival benefits. Historically, RT has been utilized as a supplement to non-D2 lymph node dissection surgery for local control (only approximately 10% receiving the standardized D2 radical resection in the Intergroup 0116 trial (7)) or R1/R2 resection, perioperative downstaging (the well-known TOPGEAR trial (8) adopted preoperative radiation and intensive ECF chemo, though it did not improve survival and there were underlying reasons behind it), adjuvant therapy for high-risk subgroup patients (e.g., pN stage ≥ N2, or nonperigastric lymph node metastases) who underwent R0 surgery performing D2 gastrectomy (the results of previous ARTIST I/II trials (9, 10) and current Qiao et al. (11) are mostly consistent), and palliative care for advanced tumors with solitary metastasis.

The current focus of the controversy has shifted from “whether neoadjuvant chemoradiotherapy (NCRT) is superior to chemo alone” to “whether specific populations need RT”. To illustrate, ongoing studies such as TOPGEAR and CRITICS-II are attempting to identify subgroups that may benefit from RT (including those with huge primary tumors and extremely heavy lymph node metastasis or GEA tumors). In addition, the Neo-CRAG study (2026 American Society of Clinical Oncology abstract number: LBA4075) compared the efficacy and safety of NCRT versus neoadjuvant chemo followed by D2 gastrectomy and adjuvant chemo in locally advanced high-risk GC/GEA (T4/N2-3). After a median follow-up of 69.7 months, the results showed dual benefits in disease-free survival (DFS) and OS. The primary endpoint, DFS, was reached (HR 0.750, P = 0.008). The 3-year DFS rate was 55.6% in the NCRT group vs. 42.4% in the chemo group, and the 5-year OS rates were 50.1% vs. 44.2% in the NCRT and chemo groups, respectively (HR 0.781, P = 0.025). However, the improvement in pathological complete response (pCR) was limited (14.8% vs. 6.2%).

### 2.2 RT in conjunction with immunotherapy

Nevertheless, outcomes for patients with locally advanced GC/GEA remain poor under conventional strategies, with postoperative pCR rates ranging from 5.6% to 29% and a 5-year OS rate of around 30%, and tumors with metastatic conditions having a median OS of 1 year (5, 6, 12–16). This is largely due to significant temporal-spatial and intertumoral-intratumoral heterogeneity, which often manifests as resistance to chemoradiotherapy and the prevalence of immunologically “cold” tumor micro-environments (immune-desert or immune-excluded phenotypes) (17, 18). Consequently, despite the synergistic but not completely developed effects of radiation combined with PD-1/PD-L1 inhibitors, it has demonstrated promising outcomes, which deserve subsequent in-depth understanding in the clinical and translational research, aiming to make an optimized combination strategy possible for patients with locally advanced GC/GEA under the premise of avoiding the additive treatment-related adverse events (AEs). At the same time, studies like TRIUNITE-05 are exploring the optimal sequential pattern of “chemo, immunotherapy, and RT” in the hope of ultimately translating an increased pCR into a survival benefit.

## 3 Dilemma and status quo

### 3.1 RT urgently needs a paradigm shift

Recently, several prominent phase III clinical trials (including the randomized Neo-AEGIS and ESOPEC trials (19, 20)) have confirmed that neoadjuvant chemoradiotherapy (NCRT) does not significantly improve OS in patients with esophageal adenocarcinoma (EA)/GEA compared with the neoadjuvant chemo (NCT) regimen (FLOT, perioperative chemo strategies initially established in western countries), prompting a reevaluation of treatment strategy. And disappointingly, certain remarkable international RCTs, such as the ARTIST series research (9, 10), the CRITICS trial (21), Zhu et al. (22), and Qiao et al. (11), have also consistently uncovered that the addition of postoperative intensity-modulated RT plus concurrent chemo did not significantly improve OS among total crowds with D2 or D1+ radical gastrectomy in GC given that the majority of recurrences appeared in distant and peritoneal or nodal basin regions outside the D2 field, but the addition of RT may reduce the local recurrence rate. In addition, the occurrence of these negative results may be related to distinctions in the staging of tumors enrolled in the study, deviations in treatment completion, outdated radiotherapy techniques, and the policy context at that time. Accordingly, despite tumor patients being well tolerated overall, this phenomenon suggests the need for a shift in the patterns from a postoperative to a preoperative RT strategy, and the poor postoperative compliance should also be taken into account. With the results disclosure of celebrated multi-center phase III clinical trials such as MAGIC, FLOT4, RESOLVE, and PRODIGY (15, 16, 23, 24), perioperative chemo (RT seems unnecessary) has become the standard for LAGC, ultimately reshaping the multidisciplinary management.

### 3.2 Shortages of chemo combined with immunotherapy

Subsequently, the Japanese randomized, phase III NExT trial (JCOG1109) (25) has provided convincing evidence and much-needed clarification to support the optimizing treatment regimen (neoadjuvant triplet CF plus D chemo) for previously untreated locally advanced esophageal squamous cell carcinoma (ESCC) patients (N = 601) who are in good condition when compared with doublet chemo and NCRT, potentially obviating the need for RT. And the ESCORT-NEO/NCCES01 study (26), initiated by Professor Li from the Cancer Hospital of the Chinese Academy of Medical Sciences, which is similar to the MATTERHORN trial (27) in resectable GC/GEA, is the leading phase III study of perioperatively implementing immunization drugs combined with chemo, aiming to compare the efficacy and safety profile of neoadjuvant camrelizumab plus chemo versus chemo alone in resectable locally advanced ESCC. The results demonstrated a significantly higher pCR rate in the intention-to-treat population with the combination regimen (28.0% vs. 4.7%, P < 0.0001), along with a manageable safety profile. However, despite the absolute improvement value reaching 12% and the controllable safety of the experimental protocol, the pCR rate with durvalumab in combination with the FLOT regimen was 19% from the MATTERHORN investigation (27). In addition, in a similar KEYNOTE-585 study (28) including T3-4 or N+ GC/GEA, even though the pCR rate for pembrolizumab-based chemo-immunotherapy increased slightly when compared to the chemo-alone group (13.4% vs. 2%), the median overall survival (mOS) was 71.8 months and 55.7 months (HR 0.86; 95% CI 0.71-1.06), indicating limited survival gains.

The focus of ASTRUM-006 (29), a randomized, double-blind, placebo-controlled, multicenter phase III investigation, is to assess the safety and effectiveness of serplulimab in conjunction with chemo for the perioperative treatment of GC as well as the adoption of ICIs alone (immune consolidation) to replace chemo following surgery. The trial aims to identify the population most likely to benefit from immunotherapy by prospectively enrolling patients with resectable GC/GEA and a PD-L1 CPS ≥ 5. Due to accumulated chemotoxicity and sluggish physical recovery, at least half of the GC patients come across it as difficult to successfully complete the intended course. This "postoperative chemo-free" strategy directly tackles these clinical pain points. And the median event-free survival (EFS) assessed by blinded independent central review was also significantly prolonged in the serplulimab group (NR vs. 52.0 months, HR: 0.67), with a 33% reduced risk of relapse; the pCR rate in the serplulimab group reached 22%, more than three times that of the control group (6%). Although neoadjuvant chemo combined with immunotherapy for GC can improve pathological outcomes, including T/N downstaging and major pathological response (MPR), there are significant differences in results between studies (the reason lies in the distinct characteristics of the enrolled patients), and the prognosis remains bleak (27, 28, 30–33). Therefore, reversing the immunosuppressive tumor microenvironment (TME) in GC/GEA to enhance immune cell infiltration represents a key research priority, particularly leveraging RT to stimulate intra- and extra-tumoral immunity.

## 4 Synergy mechanism of RT combined with immunotherapy

GC/GEA has a relatively abundant blood supply, resulting in moderate sensitivity to preoperative RT. And tumor patients have a higher degree of completion of preoperative RT due to less radiation damage and high tolerance. Meanwhile, the addition of irradiation plays a crucial role in activating the anti-tumor immune micro-environment in the human body through several molecular mechanisms. Especially, multimodality RT, which integrates low-dose radiotherapy (LDRT) with stereotactic body radiotherapy (SBRT), has the potential to enhance systemic antitumor immunity through the release of tumor antigens and remodeling of the immune micro-environment.

**Figure 1:**
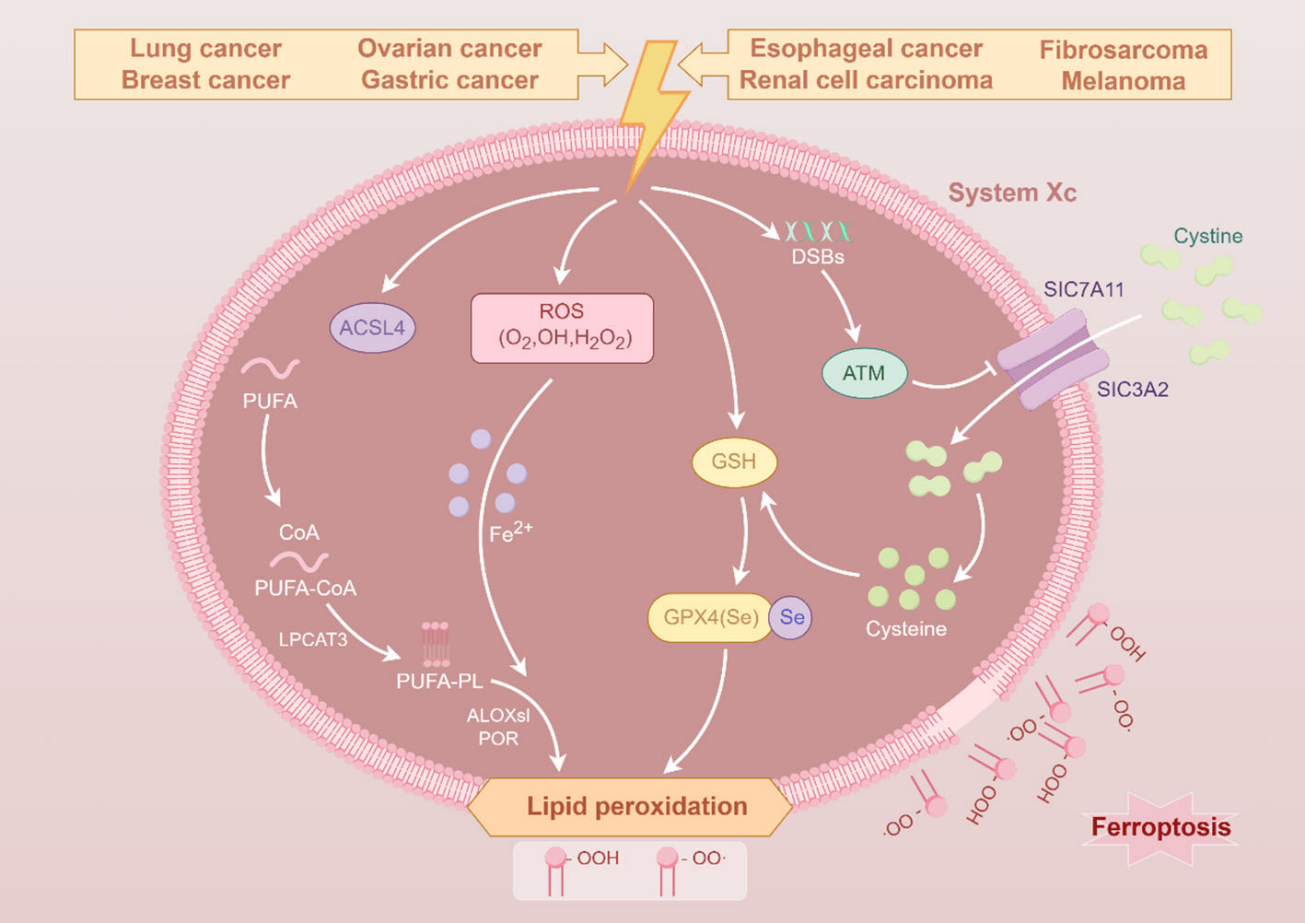
Concise mechanistic diagram of RT-induced ferroptosis. **Figure 1** was completed by Figdraw platform, for which all authors are grateful.

Firstly, it induces immunogenic cell death (ICD), which is similar to certain cytotoxic agents, leading to the release of tumor-specific antigens and immunogenic cytokines (e.g., IFN-I) that stimulate clonal expansion of tumor-specific immune cells (e.g., cytotoxic T lymphocytes and natural killer cells), thereby promoting recognition and elimination of tumor cells (34–36). Therefore, the ICD process is accompanied by increased release of multiple antigens and damage-associated molecular patterns (DAMPs), which can increase the expression of tumor-associated antigens (TAAs), thereby then participating in the activation of immune signaling pathways and promoting anti-tumor immunity. And RT could also induce lipid peroxidation and ferroptosis through at least three parallel pathways within tumors, including overproduction of ROS, upregulation of ACSL4, and depletion of GSH levels (as shown in **Figure 1**). Likewise, SLC7A11 or GPX4 inhibitors could increase the radiosensitivity of tumors by enhancing RT-induced iron death and inhibiting the overexpression of SLC7A11 or GPX4 in acquired radioresistant malignancies. Therefore, by targeting iron death, radiation has become a viable method to combat the emergence of drug resistance to the traditional treatment of tumors.

Secondly, several studies have confirmed that the cyclic Guanosine Monophosphate (GMP) and Adenosine Monophosphate (AMP) synthase-stimulator of interferon genes (cGAS-STING) signaling pathway plays a pivotal role in the innate and adaptive immune responses of autoimmune diseases, antivirals, and antitumors (37–41). Another key mechanism by which RT promotes an anti-tumor immune response is activating the cGAS-STING pathway and triggering a type I interferon cascade that enhances immune activity (42, 43). Therefore, cGAS-STING has long emerged as a promising drug target and a research hotspot in the field of anti-tumor immunotherapy and may be leveraged to provide a new anti-tumor therapy that synergistically sensitizes with RT in the coming years.

Last but not least, several studies have also demonstrated that RT can enhance the infiltration of CD8+ and CD4+ T lymphocytes and their capacity to recognize neoantigens from tumor cells by up-regulating the expression of MHC-I molecules on the surface of tumors, promoting the cross-presentation process of tumor antigens by T cells (44–46). And RT can stimulate tumor cells to release pro-inflammatory mediators and chemokines, such as CXCL9, CXCL10, and CXCL16, which could promote the infiltration of dendritic cells, macrophages, and T lymphocytes and increase their abundance, thereby effectively enhancing tumor-associated killing activities (47–49). Moreover, RT can also indirectly up-regulate the expression level of immune checkpoint proteins such as programmed cell death protein 1 (PD-1) or its ligand 1 (PD-L1) on the surface of immune cells or tumor cells via increasing interferon γ signaling, which makes it one of the potentially effective options to improve the efficacy of ICIs (50, 51). Collectively, these mechanisms synergistically enhance the host immune system’s capability to identify and eliminate tumor cells.

## 5 Promising clinical trial evidence of combining RT with immunotherapy

As ICIs combined with chemo protocols have become the first-line treatment standard for advanced GC/GEA (52), along with the confirmed synergy effect of RT and ICIs in previous landmark clinical trials (e.g., the KEYNOTE-A18 study (53) in locally advanced cervical cancer, the PACIFIC trial in unresectable stage III non-small cell lung cancer (54, 55), and the ADRIATIC trial (56, 57) in limited-stage small cell lung cancer), concurrent NCRT combined with ICIs in the neoadjuvant setting for locally advanced GC/GEA has further fueled interest and begun to be initially explored (the specific research is shown in **Table 1** (58–65)).

**Table 1:** Details of the available phase I-III trials in patients with locally advanced GC/GEA/EC who received NCRT in conjunction with immune checkpoint inhibitors.

Trial title	Clinical trial no.	Author/country	Phase/year	Study design	Sample size	Study population	Treatment design	RT dose/fraction	pCR (%)	R0 (%)	OS	PFS/EFS/DFS
PROCEED (58)	NCT03064490	Pooja Karukonda /USA	II/2023	Single-arm	35	cT1-3N0-2M0; EA/GEA/GC	NCRT (carboplatin and paclitaxel) plus pembrolizumab 200 mg*3 cycles→S→pembrolizumab 200 mg*3 cycles	45 Gy /1.8 Gy /25 F	35.7% (10/28) MPR: 50% (14/28)	97% (27/28)	NA	NA
SHARED (59)	ChiCTR1900024428	Baorui Liu /China	II/2023	Single-arm	34	cT3N2-3M0, cT4aN+M0, or cT4bNanyM0; GC/GEA	Induction chemo (S-1, nab-PTX) plus sintilimab 200 mg→NCRT (nab-PTX) plus sintilimab 200 mg*2 cycles→consolidation chemo plus sintilimab 200 mg→S→adjuvant chemo plus sintilimab 200 mg*3 cycles	45 Gy /1.8 Gy /25 F	38.2% (13/34) MPR: 79.4% (27/34)	100% (34/34)	6m-OS: 100%; 1y-OS: 92.6%	mEFS: 21.1 m; 1y-EFS: 80.1%
Neo-PLANET (60)	NCT03631615	Yihong Sun /China	II/2022	Single-arm	36	T3-4aN+M0; GC/GEA	XELOX→NCRT (xeloda)→sandwiched by XELOX→S; camrelizumab 200 mg*5 cycles during NCRT	45 Gy /1.8 Gy /25 F	33.3% (12/36) MPR: 44.4% (16/36)	100% (33/33)	2y-OS: 76.1%	2y-PFS: 66.9%
MC1541 (61)	NCT02730546	Harry H. Yoon /USA	Ib/II/2022	Single-arm	31	cT1-3NanyM0; GEA	NCRT (carboplatin and paclitaxel) plus pembrolizumab 200 mg*3 cycles→S→adjuvant pembrolizumab 200 mg*6 cycles	41.4 Gy /1.8 Gy /23 F	22.6% (7/31); PD-L1 CPS ≥ 10: 50%	90.3% (28/31)	2y-OS: 66.3%	mPFS: 19.6 m; 2y-PFS: 48.5%
ECOG-ACRIN EA2174 (62)	NCT03604991	Jennifer R. Eads /USA	II/III/2024	RCT	275	cT1N1-3M0, cT2-3N0-2M0; EA/GEA	NCRT	41.4-50.4 Gy	21.0%	NR	NR	NR
							NCRT + nivolumab 240 mg*2 cycles		24.8%			
CheckMate 577 (63)	NCT02743494	Ronan Kelly /USA	III/2025	RCT	794	II/III; EC/GEA	NCRT→S (non-pCR)→nivolumab 240 mg*8 cycles followed by nivolumab 480mg (not more than one year)	41.4-50.4 Gy (about 64% of patients)	NA	NR	mOS:51.7 m; 5y-OS: 46%	mDFS: 21.8 m
ASTRO Oral 168 (64)	NCT04061928	Rongxu Du /China	II/2024	Single-arm	43	T3-T4/N+M0; GEA (siewert II/III)	NCRT (oxaliplatin and S-1) plus toripalimab 240 mg*4 cycles→S→adjuvant SOX chemo plus toripalimab 240 mg*4 cycles	45-50 Gy /1.8-2 Gy /25 F	37.9% (21/29); MPR: 72.4% (21/29)	100%	2y-OS: 86%	2y-DFS: 89.7%
ASCO Abstract 4034 (65)	NCT03490292	Nataliya Uboha /USA	I/II/2022	Single-arm	22	T1N1M0, T2-3N0-2M0; EA/GEA	NCRT (carboplatin and paclitaxel) plus avelumab 10 mg/kg*3 cycles→S→adjuvant avelumab 10 mg/kg*6 cycles	41.4 Gy /1.8 Gy /23 F	26%	79% (15/19)	NR	NR

GC, gastric cancer; GEA, gastroesophageal junction adenocarcinoma; EC, esophageal carcinoma; OS, overall survival; RT, radiotherapy; pCR, pathological complete response; NCRT, neoadjuvant chemoradiotherapy; EA, esophageal adenocarcinoma; chemo, chemotherapy; PD-L1, programmed cell death protein ligand 1; MPR, major pathological response; HR, hazard ratio; CPS, combined positive score; TAP, tumor area positivity; m, month; mOS, median overall survival; mDFS, median disease-free survival; mPFS, median progression-free survival; mEFS, median event-free survival; RCT, randomized controlled trial; S, surgery; ASCO, American Society of Clinical Oncology; ASTRO, American Society for Radiation Oncology Annual Meeting; NR, not reported; NA, not applicable..

### 5.1 Neo-PLANET trial

The Neo-PLANET trial (60) (NCT03631615), a single-center phase II study conducted by Chinese researchers, evaluated the efficacy and safety of camrelizumab combined with concurrent chemoradiotherapy in locally advanced GC/GEA. 36 patients with cT3-4N M0 resectable tumors received XELOX chemo, NCRT (capecitabine, 45 Gy/25 fractions), and additional XELOX before surgery, along with camrelizumab throughout the treatment period. All patients completed neoadjuvant therapy and were included in the full analysis set. Thirty-three patients (91.7%) underwent surgery, all achieving R0 resection. The pCR rate was 33.3% (12/36) (95% CI, 18.6-51.0), and 44.4% (12/36) (95% CI, 27.9-61.9) achieved a major pathological response (MPR). Moreover, 77.8% (28/36) of cases had negative lymph nodes on postoperative pathology, correlating with improved clinical outcomes and underscoring the systemic immunomodulatory effects of neoadjuvant RT in GC/GEA. Treatment tolerability was acceptable, with grade III/IV AEs occurring in 80.6% (29/36) of patients, among which lymphopenia was most common (75.0%, 27/36). The *post-hoc* subgroup analyses showed that the 2-year progression-free survival (PFS) and OS rates were 60.0% and 73.1% in GEA patients and 75.5% and 80.2% in patients with GC, respectively. As a result, the long-term results of this trial are highly anticipated.

### 5.2 SHARED study

Similarly, the SHARED study (59) (ChiCTR1900024428) included 34 GC/GEA patients who received preoperative chemo-immunotherapy for induction and consolidation, along with sintilimab combined with CRT (IMRT comprised 45 Gy in 25 fractions of 1.8 Gy over 5 weeks). All patients underwent surgery, with complete R0 resection and a pCR rate of 38.2% (13/34). Grade ≥3 AEs occurred in 50% (17/34) of patients. In terms of survival benefits, this study’s 1-year event-free survival (EFS), 6-month, and 1-year OS rates were 80.1%, 100.0%, and 92.6%, respectively. In contrast, for patients with GEA receiving NCRT, the CROSS and POET studies (5, 6) revealed 1-year OS rates of 79.8% and 75%, respectively. Besides, patients with attained pCR had longer median DFS than those with non-pCR (20.9 vs. 11.1 months), consistent with a previous discovery (6).

### 5.3 MC1541 trial

However, MC1541 was a phase Ib/2 trial aiming to investigate whether pembrolizumab-containing trimodality therapy could enhance the outcome in GEA. The results showed mPFS was 19.6 months in the MC1541 cohort versus 14.6 months in the propensity-score-matched cohort who received standard NCRT (P = 0.409; 2-year PFS rates: 48.5% vs. 32.2%, HR = 0.79), while mOS was not reached for both groups. The high prevalence of unfavorable tumor characteristics in this cohort, such as clinical node positive (87%) at baseline and signet-ring cell histology (19%) through pathology specimen, both of which have been associated with a poor prognosis in this cancer, may have contributed to this.

### 5.4 ASCO 2022 and oral 168 from ASTRO

According to the American Society of Clinical Oncology (ASCO) meeting in 2022, 22 patients, including those with locally advanced EA/GEA, were enrolled in this two-part phase I/II study (65) between June 2018 and October 2021. During the trial, 19 patients had effective resections. Five patients (26%) showed excellent pCR upon resection (3/16 adenocarcinoma, 2/3 squamous cell). Therefore, compared with adenocarcinoma, squamous cell carcinoma shows superiority in tumor shrinkage and downgrading, while it is limited by design deficiencies, including small sample size and shortage of long-term results. Additionally, the toxicity profile of avelumab and CRT together was tolerable, which has similarities to the findings of the Oral 168 (64) from ASTRO (American Society for Radiation Oncology), with the pCR rate excellently reaching 37.9% (21/29) and the 100% R0 resection rate. Moreover, the survival outcome is also impressive, with a 2-year OS rate of 86% and a 2-year DFS rate of nearly 90%. However, although NCRT combined with ICIs has shown promising short-term efficacy and acceptable tolerability, further validation through large-sample studies and longer follow-up is necessary.

### 5.5 CheckMate 577 study

The global, double-blind, placebo-controlled phase III CheckMate 577 study (63) (identifier: NCT02743494) was designed to evaluate the clinical value of nivolumab adjuvant therapy in EC/GEA patients with the presence of pathological residual lesions after NCRT compared to placebo. The median follow-up was 78.3 months (range: 60.1 to 96.6), and the adjuvant nivolumab group continued to show a DFS advantage (HR: 0.76, 95% CI 0.63 to 0.91). And the mOS of the nivolumab group outperformed that of the placebo group (51.7 months vs. 35.3 months), but there was no statistical difference (HR: 0.85, P = 0.106), as well as the results of the 5-year OS rate (46% vs. 41%), highlighting the necessity to seek reliable biomarkers to enrich effective populations for ICIs delivery. Additionally, based on this pioneering research, nivolumab has been approved by the U.S. Food and Drug Administration (FDA) for adjuvant treatment in patients with EC or GEA with residual tumors (not yet reaching pCR) after NCRT.

### 5.6 Rapid oral - 2026 ASCO

The multicenter, open-label, phase IIb TERRIFIC trial (NCT05687357) evaluated neoadjuvant tislelizumab plus NCRT versus NCRT or chemo alone in patients with locally advanced GC/GEA (stage III–IVa). A total of 87 treatment-naïve patients were randomized (2:2:1) to arm A (tislelizumab 200 mg + RT with 45 Gy + SOX or S1 plus nab-PTX), arm B (NCRT), or arm C (chemo) for 3 cycles. The primary endpoint was pCR in the intent-to-treat population. Among 60 patients who underwent surgery, pCR rates were 34.8% in arm A, 25% in arm B, and 0% in arm C. MPR rates were 65.2%, 62.5%, and 15.4%, respectively. Grade ≥ 3 treatment-related adverse events occurred in 14.3% (arm A), 9.1% (arm B), and 10.5% (arm C). Hence, the addition of tislelizumab to NCRT significantly improved pCR and MPR compared with NCRT or chemo alone, with acceptable and manageable toxicity. Longer follow-up is warranted to confirm OS gains.

## 6 Biomarker deficiency and navigating reliable biomarkers for combination treatment regimens

The term “biomarker” refers to substances that are either created by tumor cells or formed by the body’s reaction to tumors throughout their presence and progression. These molecules can be identified using biochemical techniques. Various molecules, including proteins, hormones, enzymes, polysaccharides, and others, are crucial for tumor diagnosis, categorization, prognostic evaluation, and therapy monitoring. Tumor markers can be detected non-invasively or minimally invasively, which makes them popular in therapeutic settings. As they not only assist radiation oncologists in identifying cancers early on but also in assessing the effects of treatment and forecasting disease recurrence, tumor markers have emerged as a valuable complementary tool in tumor diagnosis and treatment. Thus, exploring biomarkers associated with combination therapy and screening potential beneficiaries may have also become the emphasis of perioperative treatment. And future research also needs to refine the biomarker discussion to acknowledge the heterogeneous predictive value of tumor cell PD-L1, tumor mutational burden (TMB), and immune infiltration score across trials for LAGC.

### 6.1 Tumor cell PD-L1 expression

The relationship between the expression level of PD-L1 in GC/GEA and therapeutic efficacy remains controversial and is still under ongoing investigation. The results from the SHARED study (59) showed that GC/GEA patients with tumor cell PD-L1 combined positive score (CPS) ≥ 5 had a higher pCR rate than patients with PD-L1 CPS < 5 (63.6% vs. 28.6%, P = 0.072) after completing immunotherapy combined with NCRT. Nevertheless, within the context of this study, PD-L1 CPS or tumor area positivity (TAP) score may not be associated with improved survival (DFS and OS), likely due to the limited and immature survival data. This issue is well exemplified by the coincidental ASTRUM-006 study (29), which demonstrated event‑free survival (EFS) benefits for a PD‑L1 CPS ≥ 5 in patients with resectable, locally advanced GC or GEA. Besides, the DANTE trial (32) showed that in the case of high expression of PD-L1, the tumor regression of the atezolizumab combined with the FLOT chemo group was more pronounced compared with the chemo group for resectable esophagogastric cancer. And in the MC1541 trial (61), the pCR rate was up to 50% for the baseline PD-L1 CPS ≥ 10 population (50.0% (4/8) vs. 13.6% (3/22); P = 0.046). Regrettably, numerically, but not statistically, a greater pCR rate and longer OS (2-year rates 100.0% vs. 61.8%; P = 0.126) were linked to tumors with a PD-L1 cut-off point > 1% (vs. < 1%). The phase 2 Neo-PLANET study (60) analogously demonstrated that the indicator of PD-L1 CPS at cut-offs of 1, 5, or 10 failed to predict the tumor response of neoadjuvant therapy. It should be mentioned that considering the limited sample size, the research may not have enough statistical power. Thus, the predictive value of tumor cell PD-L1 expression level for pCR remains unresolved.

### 6.2 Immune infiltration analysis

In prespecified exploratory biomarker analysis, the SHARED study has proven that enhanced levels of infiltrating CD3+ T-cells, CD4+ T-cells, CD56+ natural killer cells consisting of CD56^bright^ and CD56^dim^ subtypes, and higher M1/M1 + M2-like macrophage infiltration in stroma at baseline are associated with pCR in those patients failing to achieve pCR. Thus, higher levels of immune cell infiltration might be a good predictor of the response to immunotherapy plus NCRT in locally advanced GC/GEA.

### 6.3 Tumor mutational burden

TMB and mismatch repair deficiency (dMMR)/microsatellite instability-high (MSI-H) have also been thought to guide the efficacy assessment of ICIs for gastrointestinal tumors; the subgroup analysis results of GC from the KEYNOTE-061 study (66, 67) showed that patients with a high TMB level treated with ICIs derived significantly greater objective remission rate and OS benefits than those with a low TMB level. And based on the preliminary results of the Neo-PLANET (60) investigation, somatic mutation analysis using whole-exome sequencing of biopsy specimens revealed a considerably greater pCR rate in patients whose pretreatment TMB≥median level (4.04 mutation/Mb) than in those whose TMB<median level. And pCR was attained by all 6 patients with intestinal type and above average TMB. Both the NEONIPIGA and INFINITY trials (68, 69) have been proven to clarify the efficacy and safety of perioperative anti-cytotoxic T-lymphocyte-associated protein 4 (CTLA-4) and anti-PD-1 or PD-L1 antibodies in patients with dMMR/MSI-H locally advanced gastric and gastroesophageal junction adenocarcinoma (the pCR rate is as high as 60%). However, since the OS rate of such dMMR-type patients after the operation is still relatively high, non-surgical methods should be regarded as tentative at present. Furthermore, the combined effect of RT and immunotherapy on this type of tumor remains unknown.

### 6.4 Other unproven potential markers

Tumor-intrinsic factors (e.g., the tumor micro-environment and its components), such as higher levels of PD-L1-expressing extracellular vesicles (EV) in plasma that may identify additional responders (the MC1541 trial demonstrated a likelihood of a higher pCR rate despite low PD-L1 expression of tumor cells), are also limited by small samples, thus deserving further exploitation. In addition to the aforementioned biomarkers, Epstein-Barr virus positivity, mismatch repair proteins, tumor-specific MHC-II expression, human epidermal growth factor receptor 2 (HER-2) status, tumor-infiltrating lymphocytes, and copy number alterations may possibly be considered to be related to the treatment activity of ICIs (70), but they still need to be confirmed by clinical research in the context of locally advanced GC/GEA/EC. And although pCR has been regarded as a prognostic surrogate at the level of international standardized clinical trials, future large-scale trials will possess a longer follow-up to confirm the final OS benefits and are warranted to explore additional novel biomarkers for response prediction (71).

## 7 Conclusions and future directions

Recent results from the PREACT randomized controlled trial (NCT03013010) (72) may suggest the applications of advanced RT techniques (IMRT, VMRT, or proton with the unique physical advantage of “Bragg Peak” dosage) and the rationality of RT target design to avoid damage to non-tumor immune cells, which is proven by the initial outcomes of the comparable mRCAT-III study from the (77 cases of microsatellite-stable cT3-4N0/+M0 rectal cancer patients adopting involved field irradiation harvests about a 60% pCR rate). Irradiation of the area (the extent of irradiated positive tumor volume) and high-risk regional lymphatic drainage (e.g., lymph node stations Nos. 1-6, No. 7, No. 8a, No. 8p, No. 9, No. 10, No. 11p, No. 12, and No. 16, depending on the site of the primary focus and considerations of radiation oncology physicians); and the fractionation pattern (dosage/fraction) of RT (including personalized adaptive hypofractionated radiation therapy), as well as the sequence in which RT is combined with immunotherapy to avoid the “dark side” of RT and protect surrounding organs at risk, may also have a large impact on treatment outcomes due to whether dissimilar RT modes (the eagerly anticipated SBRT or LDRT) could transform “cold” tumors into “hot” ones and consequently boost systemic anti-tumor immunity to maximize its power (73, 74). Also, the moderate inherent radiosensitivity, deviation in the positions of human organs, and larger radiotherapy field design for locally advanced GC constitute poor local control (multiple surrounding organs are at risk, leading to an insufficient radiation dose), which could readily lead to damage to the immune system, and this requires attention from radiation oncologists.

Chemo-immunotherapy combined with RT has shown promise in the field of locally advanced GC/GEA and is gradually becoming one of the significant exploratory directions. However, there are also contradictions between studies. For example, the ECOG-ACRIN EA2174 trial (62) followed the conventional proposal (carboplatin and paclitaxel) of the CROSS study (5), and the RT dose was set between 41.4 Gy and 50.4 Gy, whose purpose is to investigate whether adding nivolumab can further improve pCR in patients with locally advanced EA/GEA. In contrast to expectations, the addition of ICIs to NCRT yielded no significant increase in pCR rate relative to NCRT alone (24.8% vs. 21.0%), highlighting the uncertainty about appropriate preoperative protocols and the need for optimal patient stratification based on molecular and pathological tumor typing.

Currently, most groundbreaking clinical studies (e.g., the PANDA trial) for GC/GEA are still carried out in the whole population to overcome the above-mentioned limitations (52, 75); for one thing, to ensure the width of enrolled groups, and for another, it also reflects that ideal immunoreaction biomarkers are still vacant, and the potential beneficiary population still needs to be further defined. In follow-up studies, how to screen the beneficiaries of immunotherapy and identify the best combination strategy among numerous treatment options still poses a huge challenge for both the study designers and the clinicians who practice the treatment plan. Although neoadjuvant immunotherapy for GC has yielded some encouraging results from small-scale exploratory trials (**Table 2** displays the excellent investigations), the utilization of ICIs should be cautious owing to the possibility of severe immunotherapy-related toxicities in clinical practice.

**Table 2:** Incorporating immunotherapy into perioperative chemo regimens retrieved from PubMed for resectable GC/GEA.

Trial title/year	Clinical trial No.	Author/country	Inclusion criteria	Study design	Sample size (EG/CG)	Treatment design (EG *vs.* CG)	pCR (%)	OS	EFS
ASTRUM-006/2026 (29)	NCT04139135	Shen L/China	Ages 18-70; PD-L1 CPS ≥ 5; resectable locally advanced GC/GEA	Randomized, double-blind, multicenter, phase III	588 (292/296)	Neoadjuvant toripalimab and SOX (3 cycles) followed by postoperative adjuvant toripalimab (up to 17 cycles) vs. neoadjuvant placebo and SOX chemo (3 cycles) followed by postoperative adjuvant SOX (5 cycles)	22% vs. 6%	NR	mEFS: NR vs. 36 m (HR: 0.73, P = 0.015), 3y-EFS: 57% vs 49%
NEOSUMMIT-01/2026 (30)	NCT04250948	Nie RC/China	Surgically eligible patients (aged 18–75, ECOG PS 0–1) with locally advanced (cT3–4aN+M0) GC/GEA	Phase II, open-label RCT	108 (54/54)	Perioperative SOX/XELOX (8 cycles) with toripalimab, then toripalimab maintenance (≤ 6 months) vs. perioperative SOX/XELOX (8 cycles)	22% vs. 7% (P = 0.030)	3y-OS: 81% vs. 72% (HR: 0.45, P = 0.036)	3y-EFS: 75% vs. 56% (HR: 0.51, P = 0.044)
DANTE/IKF-s633/2024 (32)	NCT03421288	Lorenzen S/Germany	Surgical candidates with locally advanced (≥cT2/N+M0) GC/GEA, aged ≥18, and with ECOG PS 0–1	Phase II/III, open-label RCT	295 (146/149)	8 cycles of FLOT with concurrent atezolizumab, followed by 8 cycles of atezolizumab maintenance vs. 8 cycles of FLOT chemo	24% vs. 15% (P = 0.032)	NR	NR
KEYNOTE-585/2024 (28)	NCT03221426	Shitara K/Japan	Treatment-naïve, surgically eligible patients with locally advanced G/GEA (T3+ or N+), aged ≥18, with ECOG 0–1 and a life expectancy of ≥ 6 months	Phase III, placebo-controlled, double-blind RCT	804 (402/402)	Perioperative (3 + 3 cycles) cisplatin-based chemo with pembrolizumab, then 11 cycles of pembrolizumab vs. perioperative (3 + 3 cycles) cisplatin-based chemo with placebo, then 11 cycles of placebo	14% vs. 3% (P <.0001)	mOS: NR vs. 56 m, 5y-OS: 55% vs. 49% (HR: 0.86, 95% CI: 0.71 to 1.03)	mEFS: 47 m vs. 27 m, 5y-EFS: 48% vs. 38%(HR: 0.80, 95% CI: 0.67 to 0.95)
					203 (100/103)	4 neoadjuvant/adjuvant cycles of FLOT plus pembrolizumab, then 11 cycles of pembrolizumab maintenance vs. 4 neoadjuvant/adjuvant cycles of FLOT plus placebo, then 11 cycles of placebo maintenance			
MATTERHORN/2025 (27)	NCT04592913	Janjigian YY/USA	≥18 years old with newly diagnosed, resectable GC/GEA (stage II-IVA), have an ECOG PS of 0-1, possess adequate organ function, provide a tumor sample for PD-L1 evaluation, and have received no prior anticancer treatment	Phase III, randomized, double-blind, placebo-controlled study	948 (474/474)	Perioperative FLOT (4 cycles, q4w) with durvalumab, followed by durvalumab maintenance (10 cycles) vs. perioperative FLOT (4 cycles, q4w) with placebo, followed by placebo (10 cycles)	19% vs. 7%	mOS: NR vs. NR, 3y-OS: 69% vs. 62% (HR: 0.78, 95% CI: 0.63 to 0.96, P = 0.021)	mEFS: NR vs. 32.8 m, 2y-EFS: 67% vs. 59% (HR: 0.71, 95% CI: 0.58 to 0.86, P < 0.001)
ATTRACTION-5/2024 (31)	NCT03006705	Kang YK/Korea	Patients aged 20-80 years with pathological stage III GC/GEA (post D2+ gastrectomy), ECOG PS 0-1, and available tumor tissue for PD-L1 analysis	Phase III, randomized, double-blind, placebo-controlled, multi-center trial	755 (377/378)	Patients were randomly assigned in a 1:1 ratio to receive either nivolumab (nivolumab 360 mg, q3w) plus chemo or placebo plus chemo	NA	NR	3y-RFS: 68% vs. 65% (HR: 0.90, P = 0.44)
NR/2024 (76)	NCT04341857	Ning L/China	Eligible patients with stage cT4 and/or cN+M0 GC/GEA, age 18-75 years and ECOG PS 0-1	Single-arm, open-label, Phase II trial	32	Neoadjuvant phase: sintilimab (200 mg, once every 3 weeks, for 3 cycles) combined with FLOT chemotherapy (once every 2 weeks, for 4 cycles)	17% (5/29)	3y-OS: 71%	3y- EFS: 71%

EG, experimental group; CG, control group; GC, gastric cancer; GEA, gastroesophageal junction adenocarcinoma; pCR, pathological complete response; OS, overall survival; EFS, event-free survival; RFS, recurrence-free survival; ECOG, Eastern Cooperative Oncology Group; PS, performance status; chemo, chemotherapy; HR, hazard ratio; CI, confidence interval; m, month; mOS, median overall survival; mEFS, median event-free survival; RCT, randomized controlled trial; NA, Not applicable; NR, not reported.

In conclusion, it is expected that the results of the subsequent phase II-III clinical investigations will prove the exact efficacy in the neoadjuvant background of GC/GEA so that the optimal combination mode of RT and chemoimmunotherapy via confirmed biomarkers (e.g., PD-L1 expression, minimal residual disease, and immune infiltration analysis) and patient characteristics (e.g., tumor stage, proximal or distal location, Lauren’s classification subtypes, and molecular gene profiles defined by The Cancer Genome Atlas) can make individualized treatment achievements and ultimately further enhance the OS and quality of life (QoL) benefits for patients suffering from tumors (71, 75, 77, 78). In the future, research in this field will focus more on screening the population that benefits the most (biomarker-oriented such as PD-L1 CPS and MHC-II status), optimizing the dose fraction and lymph node radiation area of RT, and subsequently exploring the synergistic effects of new drugs such as antibody-drug conjugates (ADCs), vascular targeted drugs (apatinib), and dual immunotherapy (currently with applicability restricted to rare dMMR/MSI-H subsets) (33, 79, 80).

## Glossary

GC: gastric cancer
GEA: gastroesophageal junction adenocarcinoma
OS: overall survival
RT: radiotherapy
pCR: pathological complete response
NCRT: neoadjuvant chemoradiotherapy
ctDNA: circulating tumor DNA
EA: esophageal adenocarcinoma
NCT: neoadjuvant chemotherapy
ESCC: esophageal squamous cell carcinoma
ICD: immunogenic cell death
DAMPs: damage-associated molecular patterns
TAAs: tumor-associated antigens
GMP: guanosine monophosphate
AMP: adenosine monophosphate
cGAS: cyclic GMP-AMP synthase
STING: stimulator of interferon genes
PD-L1: programmed cell death protein ligand 1
ICIs: immune checkpoint inhibitors
HER-2: human epidermal growth factor receptor-2
MPR: major pathological response
AEs: adverse events
HR: hazard ratio
EC: esophageal carcinoma
CPS: combined positive score
EV: extracellular vesicles
TMB: tumor mutational burden
dMMR: mismatch repair deficiency
MSI-H: microsatellite instability-high
CTLA-4: cytotoxic T-lymphocyte-associated protein 4
m: month
mOS: median overall survival
mDFS: median disease-free survival
mPFS: median progression-free survival
mEFS: median event-free survival
RCT: randomized controlled trial
S: surgery
QoL: quality of life
ASCO: American Society of Clinical Oncology
ASTRO: American Society for Radiation Oncology
FDA: Food and Drug Administration
EG: experimental group
CG: control group
RFS: recurrence-free survival
ECOG: Eastern Cooperative Oncology Group
PS: performance status
Chemo: chemotherapy
CI: confidence interval
NA: Not applicable
NR: not reported
ADCs: antibody-drug conjugates
TME: tumor microenvironment
LDRT: low-dose radiotherapy
SBRT: stereotactic body radiotherapy
MHC: major histocompatibility complex
